# Non-Melibiose Fermentation and Tellurite Resistance by Shigatoxigenic and Enteropathogenic *Escherichia coli* O80:H2 from Diseased Calves: Comparison with Human Shigatoxigenic *E. coli* O80:H2

**DOI:** 10.3390/vetsci12030274

**Published:** 2025-03-14

**Authors:** Rie Ikeda, Keiji Nakamura, Nicolas Korsak, Jean-Noël Duprez, Tetsuya Hayashi, Damien Thiry, Jacques G. Mainil

**Affiliations:** 1 Veterinary Bacteriology, Department of Infectious and Parasitic Diseases, Faculty of Veterinary Medicine, Center for Fundamental and Applied Research for Animals and Health (FARAH), University of Liège, B-4000 Liège, Belgium; rikeda@uliege.be (R.I.); jeannoelduprez@gmail.com (J.-N.D.); jg.mainil@uliege.be (J.G.M.); 2 Department of Bacteriology, Faculty of Medical Science, Kyushu University, Fukuoka 812-8582, Japan; nakamura.keiji.046@m.kyushu-u.ac.jp (K.N.); hayashi.tetsuya.235@m.kyushu-u.ac.jp (T.H.); 3 Food Inspection, Department of Food Sciences, Faculty of Veterinary Medicine, Center for Fundamental and Applied Research for Animals and Health (FARAH), University of Liège, B-4000 Liège, Belgium; nkorsak@uliege.be

**Keywords:** Shigatoxigenic *E. coli*, enteropathogenic *E. coli*, serotype O80:H2, melibiose fermentation, tellurite resistance, healthy cattle, zoonosis

## Abstract

Despite their prevalence in Europe, the source of contamination of humans by Attaching-Effacing Shigatoxigenic *Escherichia coli* (AE-STEC) O80:H2 remains unidentified. This study aimed to assess a procedure based on non-melibiose fermentation and resistance to tellurite to isolate AE-STEC and enteropathogenic (EPEC) O80:H2 from healthy cattle. The genome sequences of 40 calf and human AE-STEC and EPEC O80:H2 were analyzed: (i) none harbored the *mel* operon, but the *70mel* DNA sequence instead; (ii) the *ter*-type 1 operon was detected in 16 EPEC and *stx1a* or *stx2a* AE-STEC, while no *ter*-type 1 operon was detected in the remaining 24 EPEC and *stx2d* AE-STEC. The 21 calf AE-STEC and EPEC O80:H2 were tested phenotypically: (i) none fermented melibiose on melibiose-MacConkey agar plates; (ii) ten of the 11 *ter*-type 1-positive strains had Minimal Inhibitory Concentrations (MIC) ≥ 128 µg/mL to potassium tellurite; (iii) conversely, the ten *ter*-negative strains had MIC of two µg/mL. Accordingly, enrichment broths containing two µg/mL of potassium tellurite and inoculated with one high MIC (≥256 µg/mL) *stx1a* AE-STEC O80:H2 tested positive with the O80 PCR after overnight growth, but not the enrichment broths inoculated with one low MIC (two µg/mL) EPEC. Nevertheless, neither AE-STEC nor EPEC O80:H2 were recovered from 96 rectal fecal samples collected from healthy cattle at one slaughterhouse after overnight growth under the same conditions. In conclusion, this procedure may help to isolate *stx1a* and *stx2a* AE-STEC and EPEC O80:H2, but not *stx2d* AE-STEC that are tellurite sensitive, and new surveys using different procedures are necessary to identify their animal source, if any.

## Simple Summary

Although healthy cattle is the main reservoir of Attaching-Effacing Shigatoxigenic *Escherichia coli* (AE-STEC), the source of contamination of humans by AE-STEC O80:H2 remains unidentified, due in part to the absence of specific selective growth methodology. The aim of this study was to assess a procedure based on non-melibiose fermentation and resistance to tellurite to isolate AE-STEC and enteropathogenic (EPEC) O80:H2 from healthy cattle. If the 40 calf and human AE-STEC and EPEC O80:H2 did not harbor the *mel* operon, only 16 *stx1a/stx2a* AE-STEC and EPEC O80:H harbored one *ter*-type 1 operon. The 21 calf strains were further tested phenotypically: none fermented melibiose while 10 of the 11 *ter*-type 1-positive strains had Minimal Inhibitory Concentrations (MIC) ≥ 128 µg/mL. In contrast, the 10 *ter*-negative strains had MIC of 2 µg/mL. Accordingly, enrichment broths containing two µg/mL of potassium tellurite and inoculated with one high MIC (≥256 µg/mL) AE-STEC tested positive with the O80 PCR after overnight growth, but not the enrichment broths inoculated with one low MIC (two µg/mL) EPEC. As a conclusion, this procedure may help to isolate most *stx1a/stx2a* AE-STEC and EPEC O80:H2, but not *stx2d* AE-STEC that are not resistant to tellurite.

## 1. Introduction

Enterohemorrhagic *Escherichia* (*E.*) *coli* (EHEC) are a major cause of hemorrhagic colitis (HC) and hemolytic uremic syndrome (HUS) in humans and are also associated with diarrhea in young calves [1,2]. Since EHEC produce Shiga toxins (Stx1 and/or Stx2) and the Attaching and Effacing (A/E) lesion of enteropathogenic *E. coli* (EPEC), they are named “Attaching-Effacing Shigatoxigenic *E. coli*” or “AE-STEC” in this manuscript after these two pathogenic traits, as previously proposed [3].

The most frequent and pathogenic AE-STEC in humans belong to the following major O:H serotypes: O26:H11, O103:H2, O111:H-, O121:H19, O145:H-, and O157:H7 [1,4]. The main source of infection in humans are foodstuffs contaminated by intestinal contents of ruminants, such as cattle, that can be asymptomatic carriers in their intestines [1]. Serotypes O26:H11 and O111:H- are also most frequent amongst calf AE-STEC [2,5]. Besides these major AE-STEC O:H serotypes, minor ones can emerge from time to time and cause short-lived dramatic outbreaks [6] or long-lasting clinical rates [7].

Since 2010, AE-STEC serotype O80:H2 has been emerging in France in humans suffering not only from HC and HUS, but also from systemic infection [8,9,10]. In 2022, AE-STEC O80:H2 was the third leading cause of HUS in Europe, especially in young children and the elderly, behind AE-STEC O157:H7 and O26:H11 [11]. In parallel, AE-STEC and EPEC O80:H2 have also been increasingly isolated in Belgium from young calves (<3 months of age) with diarrhea or sepsis [12,13]. Calf AE-STEC and EPEC O80:H2 are related to human AE-STEC O80:H2 not only by their Sequence Type (ST301) and virulotypes but also in whole genome sequence-based phylogenetic analysis [13,14], showing the importance of this serotype as a putative agent of a serious zoonosis.

Although AE-STEC and EPEC O80:H2 have been sporadically isolated from healthy cattle and dairy products in some European countries [8,9,14], the actual source of contamination of humans is yet to be identified [10]. The lack of appropriate selective growth media is among the possible reasons. Recently, a piperacillin-supplemented melibiose-MacConkey (mel-MAC) agar was developed to isolate AE-STEC O80:H2 from diseased humans, as they do not ferment melibiose following the deletion of the *mel* operon, along with the insertion of a 70 bp DNA fragment (*70mel*) of unknown origin and function [15]. Since ureidopenicillins are not permitted in veterinary medicine in European Union (Implementing regulation—2022/1255—EN—EUR-Lex (europa.eu)) and the antibiotic resistance profile of *E. coli* from healthy cattle is difficult to predict, the general aim of this study was to assess non-melibiose fermentation (non-MF), as published by others [15], and tellurite resistance (TeR) to increase the isolation rate of AE-STEC and EPEC O80:H2 from healthy cattle. Although the resistance of AE-STEC and EPEC O80:H2 to tellurite is unknown, several other STEC and EPEC serotypes are indeed highly resistant to tellurite with Minimal Inhibitory Concentrations (MIC) > 16 μg/mL [16,17]. Therefore, different Te-supplemented growth media exist to specifically isolate the major O:H serotypes, like the cefixime-tellurite sorbitol MacConkey (CT-SMAC) agar for AE-STEC O157:H7 [18]. High levels of TeR of AE-STEC is linked to the presence of a *ter* operon that comprises six genes (*terA*, *terB*, *terC*, *terD*, *terE*, and *terZ*) and whose four variants (type 1–4) have been described [18,19].

The specific aims of this study were to: (i) detect the presence of the *70mel* fragment and of an intact *ter* operon in Belgian calf and human AE-STEC and EPEC O80:H2; (ii) confirm the non-MF by calf AE-STEC and EPEC O80:H2 and identify their MIC to tellurite; and (iii) perform a preliminary survey to assess these two properties to isolate AE-STEC and/or EPEC O80:H2 from feces collected from healthy cattle in one slaughterhouse.

## 2. Materials and Methods

### 2.1. Escherichia coli O80 Strains

A total of 44 *E. coli* O80 isolated in Belgium were studied for MF and TeR: 10 AE-STEC and 11 EPEC O80:H2 isolated from diarrheic < 3 month-old calves, 19 AE-STEC O80:H2 isolated from humans with (bloody) diarrhea and sometimes HUS, and four non-EPEC non-STEC *E. coli* O80:H6 and O80:H45 isolated from healthy adult cattle in slaughterhouses and in farms. All genome sequences are already available on the National Centre for Biotechnology Information (NCBI), BioProjects PRJNA606200 and PRJNA906740 [13,20,21].

The 40 calf and human AE-STEC and EPEC O80:H2 studied are classified into two main lineages (L) in a Single Nucleotide Polymorphism (SNP)-based phylogenetic tree [13]. The L1 lineage is subdivided into four sub-lineages (SL): SL1.1 with eight calf EPEC and five calf and human *stx1a* or *stx2a* AE-STEC, SL1.2 with 22 calf and human *stx2d* AE-STEC and three calf EPEC, SL1.3 with one human *stx2a* AE-STEC, and SL1.4 with 2 calf *stx2d* AE-STEC. The L2 lineage comprises two *stx1a* AE-STEC isolated from the same calf in 1987. The four *E. coli* O80:non-H2 are genetically not related to *E. coli* O80:H2 [21].

### 2.2. Genetic Studies

#### 2.2.1. MF-Encoding *mel* Operon and the *70mel* DNA Sequence

The DNA sequences of the three genes of the *mel* operon (*melA*, *melB,* and *melR*) and of the *70mel* fragment were obtained from the genome sequences of the *E. coli* K-12 MG1655 laboratory strain (BioProject accession number SAMN13412807) and of the AE-STEC O80:H2 RDEx444 strain (BioProject accession number SAMN08915508), respectively [15]. The detection of the *mel* operon and of the *70mel* DNA fragment in the 44 *E. coli* O80 strains was performed using the Basic Local Alignment Search tool for DNA comparison (BLASTN). The cut-off values were ≥90%, as much for the query coverage rate than for the percentage identity (https://blast.ncbi.nlm.nih.gov/Blast.cgi; accessed on 25 January 2023).

#### 2.2.2. Tellurite Resistance-Encoding *ter* Operon

The original DNA sequences of the six genes of the *ter* operon (*terA*, *terB*, *terC*, *terD*, *terE*, and *terZ*) were obtained from the genome sequence of the AE-STEC O157:H7 Sakai strain (Bioproject accession number SAMN01911278) [19] and of the six genes of each of the four types of the *ter* operon from the BioProject PRJDB10561 [22]: NZ_CP02355.1 (*ter*-type1); MH208235.1 (*ter*-type2); NZ_CP027591 (*ter*-type3); CP0232000.1 (*ter*-type4). The detection of the *ter* operon and of the four *ter*-types in the 44 *E. coli* O80 strains was also performed using BLASTN with the same query coverage rate and percentage identity (≥90%) as for the *mel* operon and *70mel* DNA sequence (https://blast.ncbi.nlm.nih.gov/Blast.cgi; accessed on 31 October 2023).

### 2.3. Phenotypic Assays

The 21 calf *E. coli* O80:H2 and the four bovine *E. coli* O80:non-H2, but not the 19 human AE-STEC O80:H2 were phenotypically studied.

#### 2.3.1. Melibiose Fermentation

The 25 calf and bovine *E. coli* O80 strains were streaked on MacConkey agar (Fisher Scientific, Bruxelles, Belgium) plates containing 10% D(+)-melibiose monohydrate (Thermo Scientific, Geel, Belgium), as described elsewhere [15]. MF was read after overnight growth at 37 °C.

#### 2.3.2. Potassium Tellurite Minimal Inhibitory Concentrations

**Figure 1:**
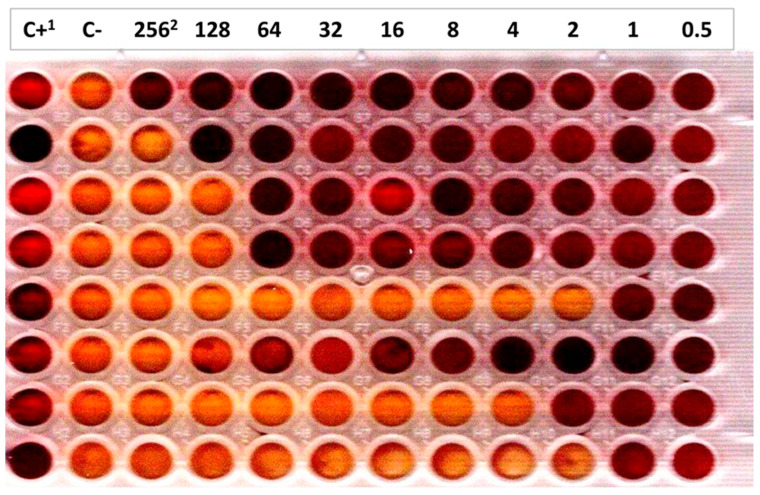
Minimal Inhibitory Concentration (MIC) determination to potassium tellurite in presence of bromocresol purple as a pH indicator. The change of color from yellow-orange to dark purple marks bacterial growth and by deduction the MIC (one strain per row). ^1^ C+: positive growth control (column 1); C-: negative growth control (column 2). ^2^ Potassium tellurite concentrations (μg/mL) in columns 3 to 12.

The MIC of the 25 calf and bovine *E. coli* O80 strains to tellurite were determined by the two-fold dilution method in 96-well micro-titre plates (VWR International, Leuven, Belgium) in Mueller-Hinton broth (VWR International, Leuven, Belgium) in presence of potassium tellurite (K_2_TeO_3_) (SIGMA-ALDRICH Chemistry, Overijse, Belgium) (256 μg/mL to 0.5 μg/mL) and of bromocresol purple as pH indicator (SIGMA-ALDRICH Chemistry, Oversijse, Belgium). After overnight incubation at 37 °C with shaking, the MIC of each strain was determined by observation of color change in the wells containing potassium tellurite (Figure 1). One *E. coli* O157:H7 strain of the Bacteriology laboratory collection harboring the six *ter* genes (*terA*, *terB*, *terC*, *terD*, *terE*, and *terZ*) and the *E. coli* K-12 DH10B laboratory strain lacking those genes, as determined by specific PCR, were the positive and negative controls, respectively [16,17,23].

### 2.4. Detection Limit of E. coli O80:H2 in Fecal Material in Presence of Potassium Tellurite

Two EPEC and AE-STEC O80:H2 with different MIC to potassium tellurite were grown overnight in lauryl-sulfate enrichment (LSE) broth for enterobacteria (VWR Life Science, Leuven, Belgium). Ten-fold dilutions were performed to obtain bacterial concentrations ranging from 10^8^ to 10^2^ CFU per mL, as previously described [24]. One mL of each ten-fold dilution was added to eight mL of LSE broth containing two µg/mL of potassium tellurite along with one g of an O80 PCR-negative fecal sample. One g of the same fecal sample was added to nine mL of un-inoculated LSE broth containing two µg/mL of potassium tellurite, as a negative control. After overnight growth at 37 °C with shaking, total DNA was extracted from two mL of each LSE broth and tested with the O80 PCR, as previously described [21].

### 2.5. Attempts to Isolate of AE-STEC and EPEC O80:H2 from Bovine Fecal Samples

One g of 96 fecal samples collected in July 2023 from the rectum of healthy adult cattle at one slaughterhouse in the province of Liège, Belgium were distributed in nine mL of two LSE broths, one containing two µg/mL of potassium tellurite, as in Section 2.4. O80 PCR-positive LSE broths and colonies were identified as previously described, except that only mel-MAC agar plates were used to streak the O80 PCR-positive LSE broths. O80 PCR-positive colonies were PCR tested for the *fliC_H2_* gene encoding the H2 antigen and O80:H2-positive colonies, if any were genome sequenced for additional typing [21].

## 3. Results

### 3.1. Genetic Analysis

All 40 calf and human AE-STEC and EPEC O80:H2 strains harbored the *70mel* DNA sequence, but not the *mel* operon, while the *mel* operon, but not the *70mel* DNA sequence was detected in the four bovine *E. coli* O80:non-H2 strains (Table 1).

The six genes of the *ter* operon were detected and identified to *ter*-type 1 in the three calf *stx1a* AE-STEC, in the five human *stx1a* or *stx2a* AE-STEC and in eight of the 11 calf EPEC O80:H2 belonging to L1/SL1.1, L1/SL1.3 and L2 (Table 1). A *ter* operon was also detected and identified to *ter*-type 3 in three of the 14 human *stx2d* AE-STEC belonging to L1/SL1.2. Conversely, no *ter* operon could be detected in any of the remaining seven calf *stx2d* AE-STEC, 11 human *stx2d* AE-STEC and three calf EPEC, belonging to L1/SL1.2 and L1/SL1.4 (Table 1). The *ter* operon was not detected in the four bovine *E. coli* O80:non-H2 strains.

### 3.2. Phenotypic Assays

None of the 21 calf AE-STEC and EPEC O80:H2 studied fermented melibiose on mel-MAC agar plates after overnight incubation at 37 °C (Table 1), in contrast to the four *E. coli* O80:non-H2 strains.

Regarding the MIC to potassium tellurite, the *E. coli* O157:H7 strain (positive control) had a MIC ≥ 256 µg/mL, while the *E. coli* K-12 DH10B laboratory strain (negative control) had a MIC of one µg/mL. Of the 11 calf *ter*-type 1-positive *stx1a* AE-STEC and EPEC, 10 had a very high MIC (≥128 µg/mL), while one EPEC had an intermediate MIC (eight µg/mL) (Table 1). Conversely, the 10 calf *ter*-negative *stx2d* AE-STEC and EPEC had low MIC (one-two µg/mL), like the four *ter*-negative *E. coli* O80:non-H2 (two-four µg/mL) and the negative control.

### 3.3. Detection Limit of E. coli O80:H2 in Bovine Fecal Material

One high MIC *stx1a* AE-STEC (MIC > 256 μg/mL) and one low MIC EPEC O80:H2 (MIC = two μg/mL) were chosen for this study. After overnight growth at 37 °C, the six enrichment broths inoculated with the highest concentrations of the *stx1a* AE-STEC tested positive with the O80 PCR. On the other hand, no positive amplification results were obtained with the seven enrichment broths inoculated with the EPEC and the negative control enrichment broth.

### 3.4. Attempts to Isolate AE-STEC and EPEC O80:H2 from Bovine Fecal Samples

After overnight growth at 37 °C in the presence or not of two µg/mL of potassium tellurite, 11 of the 96 (11.5%) enrichment broths (six with and five without tellurite) tested positive with the O80 PCR and were streaked on mel-MAC agar plates. After overnight growth at 37 °C, 13 of the 42 non-MF colonies picked-up (10 from tellurite-containing broths and three from tellurite-non-containing broths) tested positive with the O80 PCR, but none with the H2 PCR.

## 4. Discussion

Healthy cattle are the main reservoir of the major and several minor AE-STEC serotypes worldwide [1,25]. Nevertheless, the majority of the attempts using non-selective procedures to isolate AE-STEC O80:H2 from healthy cattle at slaughterhouses and from healthy cows and calves in farms have failed [8,9,14,21,26,27,28].

Even if *E. coli* O80 were identified like previously [21], no AE-STEC or EPEC O80:H2 could be recovered during this preliminary survey using a selective procedure based on non-MF and TeR. The reasons for these unsuccessful results can be several: (i) bovine AE-STEC and/or EPEC O80:H2 ferment melibiose and/or are sensitive to potassium tellurite; (ii) AE-STEC and/or EPEC O80:H2 are present in (very) low numbers in feces from healthy cattle; (iii) healthy cattle are not the primary reservoir of AE-STEC and/or EPEC O80:H2; (iv) the sizes of the samples during the different surveys are too small.

MF is not one of the reasons since the results of the genetic and phenotypic studies are straightforward and identical to those previously obtained on French human AE-STEC [15]: all tested Belgian calf and human EPEC and AE-STEC O80:H2 harbor the 70*mel* DNA fragment, but not the *mel* operon and no calf AE-STEC or EPEC ferment melibiose on mel-MAC agar plates.

In contrast, tellurite sensitivity is one reason, at least in part. Indeed, if all but one calf and human AE-STEC and EPEC belonging to L1/SL1.1, L1/SL1.3, and L2 (16 strains) harbor a *ter*-type1 operon, none of the calf and human AE-STEC and EPEC belonging to L1/SL1.2 and L1/SL1.4 (24 strains) do and only three human *stx2d* AE-STEC harbor a *ter*-type3 operon (Table 1). In agreement with the genetic results, all but one of the 11 *ter*-type 1-positive calf AE-STEC and EPEC tested have MIC ≥ 128 µg/mL, while the 10 *ter*-negative calf AE-STEC and EPEC tested have low MIC (one-two µg/mL) (Table 1). Lower MIC of *ter*-positive *E. coli*, like of the calf EPEC (MIC = eight μg/mL) have already been observed with some AE-STEC strains within the same serotype [16].

A second reason to explain the failure of isolating AE-STEC and EPEC O80:H2 from healthy cattle is that their numbers in feces do not reach the detection limit even after overnight growth, because their ecological niche would not be, in contrast to AE-STEC O157:H7, the recto-anal junction, but small or large intestinal segments, similarly to some other major serotypes, like O26:H- [25,29,30].

A third possible reason is that healthy cattle are not the primary reservoir of AE-STEC and/or EPEC O80:H2, in contrast to the major AE-STEC serotypes [1,25]. Not only other domestic and wild ruminants like sheep, goat, or deer, but also non-ruminants (pigs, wild boars), or even humans may represent their actual primary reservoir, like for some other AE-STEC serotypes [9,31].

Finally, the limited sizes of the fecal samples taken at slaughterhouses and farms, including in this study, could also explain the scarcity of positive isolation of AE-STEC and EPEC O80:H2 [8,9,14,21,26,27,28], especially since infections in humans and in calves tend to be sporadic [8,9,12,13].

Whatever the actual reason, these results imply that potassium tellurite cannot be used as a selective agent for the isolation of the great majority of AE-STEC O80:H2. This assumption is supported by the observation that two μg/mL potassium tellurite-containing enrichment broths inoculated with all but the lowest CFU concentration of the high MIC (>256 μg/mL) *stx1a* AE-STEC test positive with the O80 PCR after overnight growth, while all enrichment broths inoculated with the low MIC (two μg/mL) EPEC do not.

Besides those results, an interesting parallel observation is the simultaneous presence/absence of the *ter*-type1 operon (this study) and of the *iha* gene [13] in all but two calf and human AE-STEC and EPEC (Table 1). The two exceptions are the calf *stx2d* AE-STEC of L1/SL1.4 harboring the *iha* gene but no *ter* operon. Conversely, the *iha* gene was not detected in the three human *stx2d* AE-STEC of L1/SL1.2 harboring a *ter*-type 3 operon. The Iha (after IrgA Homologue Adhesin) adhesin protein is homologous of the Iron Regulatory Gene A (IrgA) adhesin of *Vibrio cholera* and may play a role in the intestinal colonization by AE-STEC O157:H7, although this has not been definitely demonstrated yet [32,33]. This simultaneous presence/absence of the *ter*-type 1 operon and of the *iha* gene is probably linked to their association with the prophage-like SpLE-1-like element (SpLE-1), one approximately 90 kbp prophage-like element initially identified in the AE-STEC O157:H7 Sakai strain [19,22,34,35,36,37]. Future genomic and phenotypic studies would help to confirm this hypothesis and, hopefully, to understand actual reasons, such an association between genes coding for heavy metal resistance and putative virulence factor.

## 5. Conclusions

As far as AE-STEC and EPEC O80:H2 are concerned, the selective isolation procedure based on non-MF and TeR may help to isolate most *stx1a* and *stx2a* AE-STEC and EPEC, but not *stx2d* AE-STEC that are not TeR.

Nevertheless, future surveys will be worth performing using either selective with different antibiotics, or non-selective procedures with fecal samples and/or samples taken from different intestinal segments (when possible) not only of healthy cattle in slaughterhouses and farms but also of other domestic and wild animal species in European countries and even of healthy humans [31,38,39].

Finally, future genome and phylogenetic analysis of more AE-STEC and EPEC O80:H2 from calves, humans and other sources should help to decipher and understand the molecular evolution of the different (sub-)lineages of this still emerging serotype.

## Figures and Tables

**Table 1:** Melibiose fermentation and tellurite resistance of the 44 calf and *human E. coli* O80 according to their classification in (sub-)lineages in a Single Nucleotide Polymorphism (SNP)-based phylogenetic tree, and detection of the *iha* gene.

Serotype ^1^	Source ^1^ (No. Strains)	Virulotype ^1^	Melibiose Fermentation			Tellurite Resistance		(Sub-)Lineage (L/SL) ^1^	No. Strains	*iha* Gene (WGS) ^1,2^
			*mel* Operon (WGS) ^2^	70mel DNA Sequence (WGS) ^2^	Melibiose McConkey ^3^	*ter* Type Operon (WGS) ^2,4^	Te^++^ MIC (ug/mL) ^5^			
O80:H2	Calves (21)	*eaeξ*	-	+	-	+ (t1)	>256	L1/SL1.1	1	+
							256		4	
							128		2	
							8		1	
						-	2	L1/SL1.2	3	-
		*eaeξ stx1a*	-	+	-	+ (t1)	>256	L1/SL1.1	1	+
								L2 **^6^**	2	
		*eaeξ stx2d*	-	+	-	-	2	L1/SL1.2	5	-
								L1/SL1.4	2	+
	Humans (19)	*eaeξ stx1a*	-	+	ND	+ (t1)	ND	L1/SL1.1	3	+
		*eaeξ stx2a*	-	+	ND	+ (t1)	ND	L1/SL1.1	1	+
								L1/SL1.3	1	
		*eaeξ stx2d*	-	+	ND	+ (t3)	ND	L1/SL1.2	3	-
						-			11	-
O80:non-H2	Bovines	-	+	-	+	-	2–4	-	4	-

^1^ from reference [13]. ^2^ operon/DNA sequence/gene detected (+) or not (-) after Whole Genome Sequencing (WGS). ^3^ melibiose fermentation (+) or not (-) after overnight growth at 37 °C on mel-MAC agar plates; ND: not done. ^4^ + (t1): the *ter*-type 1 operon was detected with >99.9% homology; + (t3): the *ter*-type 3 operon was detected with >99.9% homology. ^5^ Te^++^ MIC: Minimal Inhibitory Concentration of potassium tellurite; ND: not done.^6^ *stx1a* AE-STEC EH2282 strain was erroneously referred to as *stx2a* in Figure 1 of reference [13].

## Data Availability

All data are contained within the article.
